# Predictors of breast cancer cell types and their prognostic power in breast cancer patients

**DOI:** 10.1186/s12864-018-4527-y

**Published:** 2018-02-13

**Authors:** Fan Wang, Zachariah Dohogne, Jin Yang, Yu Liu, Benjamin Soibam

**Affiliations:** 1 Department of Oncology, the First Affiliated Hospital of Xian Jiaotong University, Xi’an, Shaanxi Province 710061 People’s Republic of China; 2 Department of Biology and Biochemistry, University of Houston, Houston, TX 77204 USA; 3 Computer Science and Engineering Technology, University of Houston-Downtown, Houston, TX 77002 USA

**Keywords:** Breast cancer, Logistic regression, HER2 positive, Single cell sequencing

## Abstract

**Background:**

Comprehensive understanding of intratumor heterogeneity requires identification of molecular markers, which are capable of differentiating different subpopulations and which also have clinical significance. One important tool that has been addressing this issue is single cell RNA-Sequencing (scRNASeq) that allows the quantification of expression profiles of transcripts in individual cells in a population of cancer cells. Using the expression profiles from scRNASeq, current studies conduct analysis to group cells into different subpopulations using clustering algorithms. In this study, we explore scRNASeq cancer data from a different perspective. We focus on scRNASeq data originating from cancer cells pertaining to a particular cancer type, where the cell type or the subpopulation to which each cell belongs is known. We investigate if the “cell type” of a cancer cell can be predicted based on the expression profiles of a small set of transcripts.

**Results:**

We outline a predictive analytics pipeline to accurately predict 6 breast cancer cell types using single cell gene expression profiles. Instead of building predictive models using the complete human transcripts, the pipeline first eliminates predictors with low expression and low variance. A multinomial penalized logistic regression further reduces the size of the predictors to only 308, out of which 34 are long non-coding RNAs. Tuning of predictive models shows support vector machines and neural networks as the most accurate models achieving close to 98% prediction accuracies. We also find that mixture of protein coding genes and long non-coding RNAs are better predictors compared to when the two sets of transcripts are treated separately. A signature risk score originating from 65 protein coding genes and 5 lncRNA predictors is associated with prognostic survival of TCGA breast cancer patients. This association was maintained when the risk scores were generated using 65 PCGs and 5 lncRNA separately. We further show that predictors restricted to a particular cell type serve as better prognostic markers for the respective patient subtype.

**Conclusion:**

Our results show that in general, the breast cancer cell type predictors are also associated with patient survivability and hence have clinical significance.

**Electronic supplementary material:**

The online version of this article (10.1186/s12864-018-4527-y) contains supplementary material, which is available to authorized users.

## Background

A malignant group of tumor cells is comprised of distinct subpopulations. This clonal diversity is an important feature of many human tumors [1–3] and it is necessary for the evolution of the cancer cells into different subpopulations, which differ in their genetic characteristics [4–6]. This underlying difference is exhibited in external behaviors pertaining to invasion, metastasis, and the resistance to drug treatment. Therefore, it possesses a significant challenge in designing effective treatment strategies.

Single cell sequencing is emerging as one of the most important technology to understand tumor heterogeneity at the resolution of single cells [7, 8]. Single-cell DNA sequencing methods allow us to understand the diversity of copy number alterations, resolve clonal substructure and genetic lineages, identify rare mutations, characterize subpopulations of cancer cells etc. For example, a large number of rare subclonal (< 1%) mutations that may play an important role in tumor evolution and therapy resistance were identified in breast cancer [9]. Single-cell exome sequencing has been successfully applied to study clonal substructure in a muscle-invasive bladder cancer [10] and a colon cancer [11]. On the other hand, single cell RNA-Sequencing (scRNASeq) allows quantification of expression profiles of transcripts in individual cells in a population of cancer cells. Using the expression profiles, cells can be grouped into different subpopulations using clustering algorithms. For example, scRNASeq identified three distinct gene signatures in circulating tumor cells associated with metastasis [12]. In another study, scRNASeq was used to study the spread of single circulating tumor cells and circulating tumor cell clusters in metastatic breast cancer patients and mouse models [13].

One essential component to gain a comprehensive understanding of intratumor heterogeneity is identification of molecular markers, which can accurately differentiate different subpopulations of tumor cells and their clinical significance in cancer patients. To address this issue, we explore scRNASeq cancer data from a different perspective. We focus on scRNASeq data originating from cancer cells pertaining to a particular cancer type, where the cell type or the subpopulation to which each cell belongs is known. We investigate if the “cell type” of a cancer cell can be predicted based on the expression profiles of a small set of transcripts and if the set of these transcripts are associated with cancer patient survival rate. Since most of the publicly available data use few numbers of cells, we focused on a breast cancer study that used an ample number of cells originating from 6 different cell types. Here, we outline a predictive analytics pipeline to accurately predict the 6 breast cancer cell types using single cell gene expression profiles. Instead of building predictive models using the entire annotated human transcripts, the pipeline first reduces the number of predictors by filtering transcripts with low expression and low variance. Applying a multinomial penalized logistic regression process, the number of predictors is further reduced to 308 transcripts, out of which 34 were long non-coding RNAs. Tuning of predictive models on the reduced data set with these 308 predictors shows support vector machines and neural networks as the most accurate models achieving close to 98% prediction accuracies. Our study indicates that the cell type of a breast cancer cell can be expressed as dependent variable of expression profiles of a small set of transcripts. We also find that this mixture of protein coding genes and long non-coding RNAs are better predictors compared to when the two sets of transcripts are treated separately. From the 308 predictors, a signature risk score using 68 protein coding genes and 5 lncRNAs predictors is associated with prognostic survival of TCGA breast cancer patients. This association was maintained when the risk scores were generated using 68 protein coding genes and 5 lncRNAs separately. We further show that predictors restricted to a particular cell type serve as better prognostic markers for the respective patient subtype**.** This shows that in general, the breast cancer cell type predictors are also associated with patient survivability and hence have clinical significance.

## Results

### Breast cancer single cell transcriptome data

To the best of our knowledge, we found seven major studies that used single cell RNA-Seq studies pertaining to cancer. These studies were based on Glioblastoma [14], Melanoma [12], Pancreatic cancer [15], cancer cell lines [16], and breast cancer [13, 17]. However, we focused only on one scRNASeq data on breast cancer cells because this study used ample number of cells, the raw data was publicly available, and cell type information for predictive analytics was also available. The data pertaining to this study was obtained from NCBI GEO site with accession number GSE75688. The scRNASeq breast cancer data had 401 cells and originated from 6 different cell types populations: 15% estrogen receptor positive (ER+), 7% double positive (ER + HER2+), 12% BC03LN (lymph node metastasized double positive), 29% human epidermal growth factor receptor 2 positive (HER2+), 29% triple-negative breast cancer (TNBC), and 8% BC07LN (lymph node metastasized triple-negative).

**Fig. 1:**
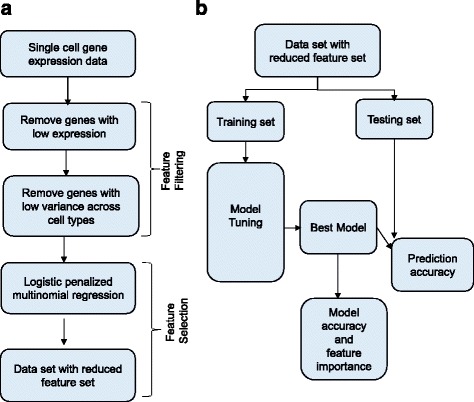
The predictive analysis pipeline. **a** Panel (**a**) show the steps involved in removing the number of genes excluded from the set of predictors for breast cancer cell type. **b** Panel (**b**) shows the basic model tuning process. The data is split into training and testing sets. Models are tuned using the training set. Optimal model is used for prediction in the testing set

The predictive analysis pipeline used in this paper is summarized in Fig. 1 and is divided into two sections. The first part of the analysis is feature filtering and feature selection to reduce the number of gene predictors to be used in building predictive models. The second part of the analysis involves tuning various predictive models for accurate prediction of cell type of breast cancer cells.

### Feature selection process retains 308 predictors

**Fig. 2:**
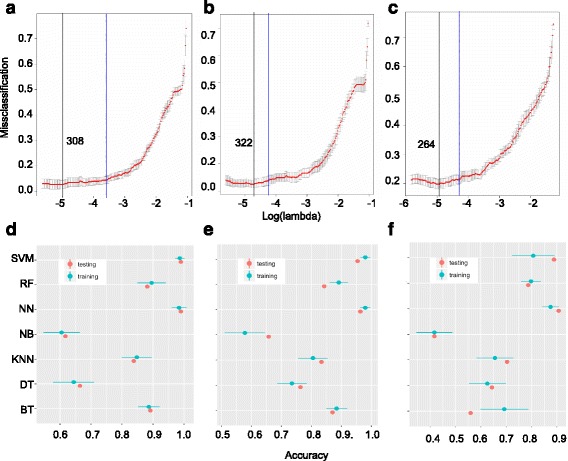
Feature selection and model tuning results. Panels (**a**), (**b**), and (**c**) show the results of applying the LASSO regression process by using both protein-coding genes, protein-coding genes only, and long non-coding RNAs only, respectively. The x-axis is the tuning parameter in log-scale and the y-axis is the 10-fold cross-validated misclassification error. The black vertical line indicates the optimal model with the lowest misclassification error, the blue vertical line indicates a lesser complex model with misclassification error within one standard error of the optimal model. The number indicated next to the black vertical line is the number of predictors selected by the optimal LASSO regression model. Panels (**d**), (**e**), and **f** show the prediction accuracy results of 7 predictive models (SVM: support vector machines, RF: random forest, NN: neural network, NB: Naïve Bayes, KNN: k-nearest neighbor, DT: decision trees, BT: boosted trees) using the reduced set of predictors from panel (**a**), (**b**), and (**c**), respectively. The prediction accuracies for training and testing sets are indicated

The original data contained the expression profiles of approximately about 50,000 genes (including protein-coding and long noncoding RNAs). Since, we are interested in finding a smaller set of transcripts which can be used as cell type predictors, it is not feasible nor appropriate to use all these genes as predictors. First, the genes which had low expression across all cells (maximum expression < 2 FPKM or fragments per kilobase of exon per million reads mapped) or the genes which didn’t have significantly high variance across the cell types (anova test, *p*-value > = 0.01) were removed. These steps reduced the number of genes in breast cancer data set to 5592 genes. This reduced data set was processed through feature selection process using a multinomial penalized LASSO logistic regression technique (Methods). Tuning this model was done by varying the regularization parameter (lambda) and it yielded an optimal classification result with classification error of 13% for lambda = 0.006898 (Fig. 2a). The feature selection process embedded in LASSO tuning selected 308 predictor genes in the optimal model, out of which 34 were long noncoding RNAs. This reduced data set containing these 308 predictors was used for subsequent analysis.

### Tuning predictive models

Since there was imbalance in the distribution of number of cells across the 6 cell types, we used the SMOTE algorithm [18] to achieve balance across the 6 groups. After this, the data set with 308 predictors was split into training (80%) and test (20%) sets. To make sure that there was no bias in the split for a particular cell type; population from each cell type contributed 80% to training and 20% to test set. Using 10-fold cross validation resampling technique, the following models were tuned by varying the respective model tuning parameters: K-nearest neighbor, decision trees, support vector machines, ensemble models (random forest and boosted trees), neural networks, and Naïve Bayes. After tuning, neural networks and support vector machines gave the best accuracies on the training and test sets (Fig. 2d). Neural network (NN) and support vector machines (SVM) showed the best predictive ability of the breast cancer cell types. NN achieved accuracies of 98.41% +/− 2.5% and 99% accuracies on training and testing data, respectively and SVM achieved accuracies of 98.66% +/− 1.55% and 99% accuracy on training and testing data, respectively (Fig. 2d). These two models were followed by Random forest, and boosted trees. K-nearest neighbors, decision trees, and Naïve Bayes performed the worst (Fig. 2d).

### Tuning predictive models with long non-coding RNAs and protein-coding genes as predictors

We noticed that the original 50,000 predictors were not all protein coding genes; some of them were long non-coding RNAs. To compare the predictive power of long noncoding RNAs and protein coding genes, we generated two different data sets from the original data set that contained 50,000 genes. One set consisted only long noncoding RNAs (lncRNA-set) and the second set consisted only protein coding genes (pcg-set). We applied the same feature selection procedure followed by penalized LASSO regression tuning step on these two data sets separately. With the pcg-set, LASSO tuning process yielded a classification error of 13% with lambda = 0.00721 with an optimal set of 322 protein-coding genes predictors (Fig. 2b). With the lncRNA-set, classification error of 21% with lambda = 0.00721 with an optimal 264 lncRNA predictors was achieved (Fig. 2c).

Model tuning using only the 322 protein-coding gene predictors yielded accuracies of 97.94% +/− 1.71% and 97.95% +/− 1.68% accuracies on the training sets by NN and SVM, respectively and 96.30% and 95.37% accuracies on the testing sets by NN and SVM, respectively (Fig. 2e). This shows that the predictive power of protein-coding genes is slightly lower compared to predictive ability of the mixture of lncRNAs and protein coding genes. However, tuning models using only 264 lncRNA predictors yielded much lower predictive accuracies (87.52% +/− 3.29% for NN and 80.78% +/− 18.40% for SVM for training sets) (Fig. 2f). These results indicate that when considered separately, lncRNAs and Protein-coding genes are weaker predictors for breast cancer cell types. However, the right mixture of protein-coding genes and lncRNAs act serve as accurate predictors for breast cancer cell types. Therefore, we focus on the original 308 predictors for further analysis.

### Clustering predictors

**Fig. 3:**
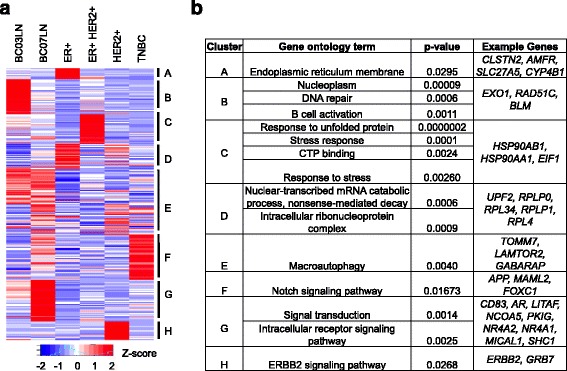
Clustering results of expression profiles of predictors. Panel (**a**) shows a heat map representing the clustered expression profiles of 308 predictors used in Fig. 2a. The cluster ID are indicated against the heat map. Panel (**b**) shows the gene ontology analysis of predictor genes belonging to the clusters in panel (**a**). Example genes belonging to the clusters are also shown

Next, we investigated the expression patterns of the 308 predictors (34 lncRNAs + 274 PCGs) across the 6 different cell types by performing HOPACH clustering using cosine dissimilarity as the distance metric. This yielded 8 optimal clusters (Fig. 3a). Interestingly 6 clusters showed a restricted expression pattern to one single cell type (Fig. 3a). Clusters A, B, C, F, G and H are restricted to ER+, BC03LN, ER+ HER2+, TNBC, BC07LN, HER2+ cell type, respectively (Fig. 3a). Cluster D is restricted to ER+ and HER2+ cells, while cluster E is restricted to BC03LN and BC07LN cells (Fig. 3a). These results show that expression of the majority of the predictors is restricted to a single breast cancer cell type.

Gene ontology analysis for the predictors in each cluster revealed enrichment of unique GO terms with no overlap between the clusters. For example, ER+ restricted cluster A predictors were enriched in Endoplasmic reticulum membrane, HER2+ restricted predictors in cluster H were associated with ERBB2 signaling pathway, lymph node metastasized double positive BC03LN restricted predictors in cluster B were enriched in DNA repair, and B cell activation, triple negative TNBC restricted predictors (cluster F) were particularly enriched in Notch signaling pathway, predictors restricted to ER + HER2+ (cluster C) were enriched in stress response, CTP binding, and response to unfolded protein (Fig. 3b).

We found that some known breast cancer markers were included in our 308-predictor set. For example, *CYP2A6*, which is known to be directly induced by estrogen in an ER-dependent manner [19], is one of the predictors and its expression is restricted to ER+ cell type (Fig. 3b). *ERBB2* gene, an important marker for HER2+ cancer patients, is also included in our predictor set and its expression is restricted to HER2+ breast cancer cell type (Fig. 3b). Androgen receptor (*AR*) is known to be a tumor suppressor in ER+ breast cancer type [20] is one of the predictor belonging to cluster G (Fig. 3b) and is not expressed in ER+ cells (Fig. 3a and b) but expressed in BC07LN (metastasized triple negative TNBC cells). This is reminiscent of the previous study that indicates some association of *AR* with prognosis of triple negative (TNBC) breast cancer patients [21]. *Hsp90* proteins overexpression has been proposed to have some role in making breast cancer cells become resistant to various stress stimuli [22] . We found that several *Hsp90* proteins (for example *Hsp90AB1*, *Hsp90AA1* etc.) were also included in the predictor set and were highly expressed in double positive ER + HER2+ cells (Fig. 3a and b). Note that the 308 predictors can discriminate the 6-different breast cancer cell types. Hence, it is possible that known key markers for a particular breast cancer cell type may not be overall good candidate for differentiating between the 6 cancer cell types. For example, the gene *ER*, which is an important marker for ER+ cells, was absent in the set of 308 predictors.

### Survival analysis of TCGA cancer patients using cell type predictors

**Fig. 4:**
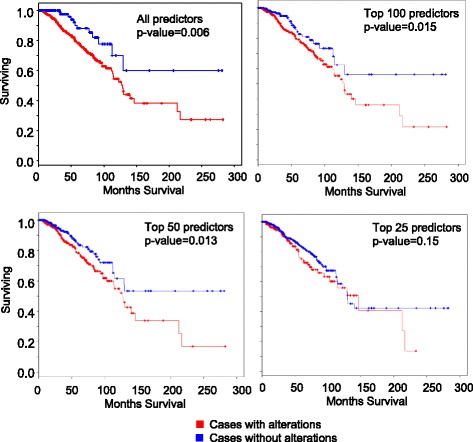
Survival curve analysis of TCGA breast cancer patients based on cancer cell type predictors. The patients were divided into two groups. One group had genetic alteration (focal amplification or deletion or mutation) in at least of one of all the predictors. The other group had none. Similarly, patients were stratified using based on the top 100, 50 ranked predictors and top 25 ranked predictors. Survival curve analysis was done using Cox hazard regression model. The statistical difference between the two groups of patients are indicated by the *p*-value

Next, we checked if the predictor genes have prognostic power in stratifying breast cancer patient risk and likelihood of survival. First, we ranked the predictors used in the neural network model using a model-independent metric (Methods) using the CARET package [23]. We gathered clinical data for 816 breast cancer patients from the cancer genome atlas project (TCGA). We found that 70% of the patients had alteration in at least of one of top 100 ranked predictors, however 82% of the patients had alteration in at least of one of the 308 predictors. Further separating the patients based on type of alteration, we found 75% (614 out of 816) patients had amplification or deletion, while 27% (221 out of 816) of the patients had mutation in at least one of the top 100 ranked predictors. We divided the patients into two groups depending on whether the patient had or no alterations in at least one of the 308 predictors. Overall survival Kaplan-Meir estimate analysis, with overall survival or event-free survival (“days to last follow up” for alive patients and “days to death” for deceased patients in the clinical data file) as the dependent variable was used to analyze the clinical data. Patients with alteration had significantly less likelihood of survival compared to the patients without alteration (Fig. 4, *p*-value = 0.0068). There was also a significant difference between the two groups of patients by using the top 100 predictors (Fig. 4, p-value = 0.015), 50 predictors (Fig. 4, p-value = 0.013). However, no significant difference (*p*-value = 0.15) was observed in the number of months of surviving when the patients were stratified using the top 25 predictors (Fig. 4, p-value = 0.15). These results suggest that the presence of alteration in top breast cancer cell type predictors can be an indicator for likelihood of survival in breast invasive carcinoma patients.

**Fig. 5:**
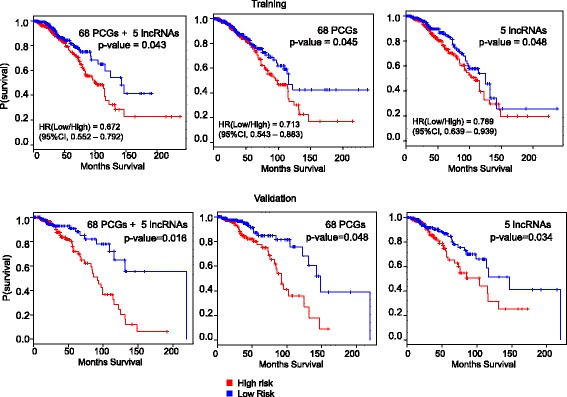
Prognostic power of breast cancer cell type predictors. The TCGA breast cancer patients were stratified into high risk and low risk patients based on the expression profiles of the breast cancer cell type predictors in TCGA patients. The best multivariate Cox proportional hazard regression model was obtained using a training set (80% of the patients) and was tested on a validation set (20% of the patients). The statistical difference between the two groups of patients are indicated by the p-value

Next, we checked if the expression signature of the predictors could be used for survival prognosis of TCGA breast cancer patients. The TCGA breast cancer patients were partitioned into training (80%) and testing (20%) data sets. Using the training data set, we identified individual cell type predictors with a prognostic power for the TCGA breast cancer patients. The log2-transformed normalized expression value of each predictor in the training set was analyzed using a univariate Cox proportional hazard regression model to assess its association with patient’s survival. We found 73 predictors (68 PCGs and 5 lncRNAs), each of which had a significant prognostic power (Fig. 5, *p*-value < 0.05). These 73 predictors were selected and their expressions were analyzed together using a multivariate Cox proportional hazard regression model. After the model was fitted, each patient was assigned a risk score as the weighted sum of log2 expression values of the selected 73 predictors (the weights were the coefficients obtained from the fitted multivariate Cox model). Based on the risk score, the patients in the training data set were divided into two groups - high risk (top one-half of signature risk score, *n* = 254 with 37 events), and low risk (bottom one-half of signature risk score, *n* = 445 with 22 events) patients. The Kaplan-Meier analysis showed a moderately significant difference in patients’ survival between the high-risk group and the low-risk group (Fig. 5, *p*-value =0.043). To validate the prognostic power of this set of 73 predictors, the patients in the testing data set were assigned a risk score using the same coefficients in the multivariate Cox model trained using the training data set. The patients in the testing set were divided into 161 low (with 17 events) and 191 high-risk (with 42 events) patients using the same risk cutoff used in the training set. Consistent with the training set, there was significant difference between the low-risk and high-risk patients in the testing data set (Fig. 5, *p*-value =0.013). In addition, the 73 predictors’ signature was further applied to the entire TCGA breast cancer dataset. As in the training and testing dataset, the signature could also classify the entire patient dataset into a high-risk group (n = 445 with 79 events) and a low-risk group (*n* = 376 with 39 events) with significantly difference in likelihood of survival (Fig. 5, p-value = 0.0106).

We further checked if the 68 PCGs and 5 lncRNAs (ENSG00000250337, ENSG00000224137, ENSG00000266088, ENSG00000238121, and ENSG00000260257) also have prognostic power when applied separately. Two separate multivariate cox models were generated with the 65 PCGs and 5 lncRNAs sets on the training data set. Following similar procedure of assigning risk scores based on weighted sum of log2 expression values followed by dividing patients into high and low risk groups, Kaplan-Meier analysis revealed moderately significant difference between the two patient groups using the 5 lncRNAs set (Fig. 5, p-value = 0.045) and 68 PCGs set (Fig. 5, p-value = 0.048). Their prognostic power was also validated using the testing data set (Fig. 5, p-value = 0.034 for 5 lncNAs and 68 PCGs set p-value = 0.002).

We further checked if prognosis improves by considering the 6 sets of predictors (identified in Fig. 3) specific to 6 cell types separately. For this purpose, instead of considering all the predictors, each of the 6 set of predictors specific to 6 cell types were analyzed using a multivariate Cox proportional hazard regression model on the entire TCGA patients (Additional file 1: Figure S1). Interestingly, all the six set of predictors significantly (all *p*-values < 10^− 6^) separated the patients into high risk and low risk patients (Additional file 1: Figure S1). This indicates that prognosis improved by considering the 6 sets of predictors specific to 6 cell types separately and the 6 set of predictors performed equally as prognostic markers (Additional file 1: Figure S1).

### Cell type specific predictors serve as better prognostic markers for respective patient subtype

**Fig. 6:**
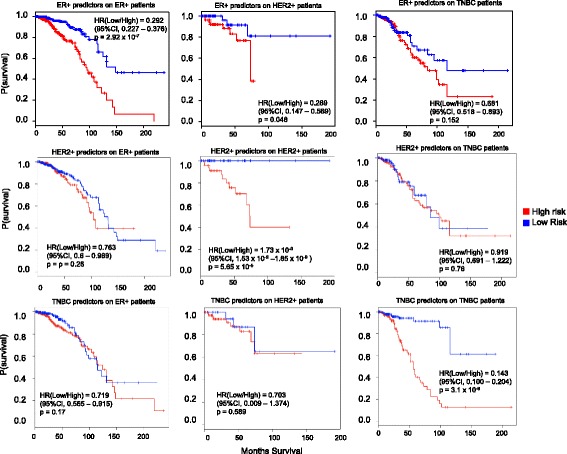
Cell type specific predictors do show prognostic potential in patients of respective subtype. Predictors whose expression restricted to ER+, HER2+, and TNBC types were considered separately. They were analyzed separately using a multivariate Cox proportional hazard regression model only on the TCGA patients belonging to the subtype representing the cell type of interest. Analysis show that ER+ cell type specific predictors show better prognostic potential in TCGA ER+ patients compared to HER2+ and TNBC patients. Similarly, HER2+ and TNBC cell type specific predictors serve as better prognostic markers in HER2+ and TNBC patients respectively. *P*-values along with hazard ratios and confidence intervals are indicated

In Fig. 5 and Additional file 1: Figure S1, we showed that the cell type predictors can serve as prognostic markers in breast cancer patients irrespective of the cancer subtype the patient belongs to. We identified 6 clusters in Fig. 3, which were specific to different types of breast cancer cells. We explored if the predictors restricted to a particular cell type showed better prognostic significance for the respective breast cancer subtype in TCGA patients. For this purpose, we only considered predictors specific to ER+, HER2+, and TNBC types and their respective TCGA subtypes. The other groups were not considered because of small number of TCGA cancer patients. First, we separated the TCGA patients into three groups: ER+, HER2+ and TNBC patients. Then the predictors specific to a particular cell type were analyzed together using a multivariate Cox proportional hazard regression model only on the TCGA patients belonging to the subtype representing the cell type of interest. After the model was fitted, each patient belonging to the subtype representing the cell type of interest was divided into two groups - high risk, and low risk (bottom one-half of signature risk score) patients. Survival curve analysis was then performed on the two groups. To compare the prognostic significance of cell type specific predictors, patients belonging to other subtypes were then assigned a risk score using the same coefficients in the fitted multivariate Cox model. The above process was done for ER+, HER2+, TNBC specific predictors. We found that predictors restricted to a particular cell type showed better prognostic significance for the respective breast cancer subtype in TCGA patients (Fig. 6). For example, ER+ predictors separated the survival rate of ER+ TCGA patients into high risk and low risk groups with higher significance (Fig. 6, *n* = 583 with 77 events, *p*-value = 2.92 × 10^− 7^) compared to HER2+ TCGA patients (Fig. 6, *n* = 110 with 12 events, p-value = 0.048) and TNBC TCGA patients (Fig. 6, 302 with 58 events, p-value = 0.681). Similarly, HER2+ predictors separated the survival rate of HER2+ TCGA patients into high risk and low risk groups with higher significance (Fig. 6, *p*-value = 5.65 × 10^− 5^) compared to ER+ TCGA patients (Fig. 6, p-value = 0.26) and TNBC TCGA patients (p-value = 0.76). TNBC predictors separated the survival rate of TNBC TCGA patients into high risk and low risk groups with higher significance (Fig. 6, p-value = 3.1 × 10^− 8^) compared to ER+ TCGA patients (Fig. 6, *p*-value = 0.17) and HER2+ TCGA patients (Fig. 6, *p*-value = 0.589). Considering the Hazard ratios of the prognostic analysis using cell type specific predictors on the TCGA patients with respective subtypes, patients with low risk most likely die at a lower rate compared to high risk patients (Fig. 6, 0.292 for ER+, 1.73 × 10^− 9^ for HER2+, and 0.143 for TNBC).

### Cell type specific predictors serve as prognostic markers for early stage breast cancers

**Fig. 7:**
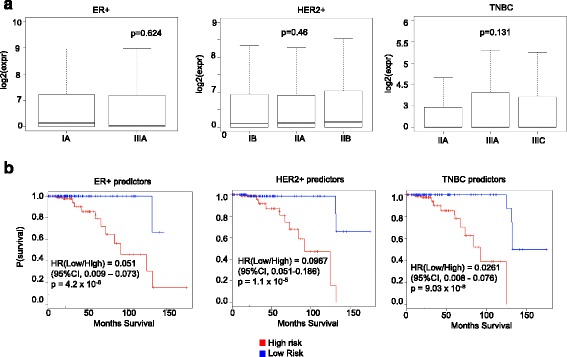
Prognostic potential of predictors in early stage breast cancer patients. **a** Expression of predictors specific to ER+, HER2+, and TNBC cell type predictors in cells at different stages. P-values are indicated in each boxplot. **b** Prognostic potential of predictors specific to ER+, HER2+, and TNBC types in stage I TCGA breast cancer patients. P-values along with hazard ratios and confidence intervals are indicated

Providing good prognosis to early stage breast cancer patients is crucial. Therefore, we further checked if the cell type specific predictors can also serve as good prognostic markers in early stage breast cancer patients. First, we validated that the single cells for each type were not restricted to a single stage, they were taken from different patients at different stages (GSE75688). ER+ cells were taken from stage I and III, HER2+ cells pertained to stages II, and I, and TNBC cells came from stages I, II, and III (GSE75688). We also tested if the cell specific predictors showed unbiased expression across cells coming from different stages. Using analysis of variance, we showed that cell type specific predictors showed no significant difference in the expression levels across the stages (Fig. 7; ER+ cells, p-value = 0.624; HER2+ cells, p-value = 0.46; and TNBC+ cells, p-value =0.131). After this, predictors specific to a particular cell type were analyzed together using a multivariate Cox proportional hazard regression model on the stage I TCGA patients. We didn’t separate the patients to the respective subtypes because this led to low number of patients in each group. We found that each cell specific predictors served as good prognostic markers for early stage breast cancer patients (Fig. 7, ER+ predictors with p-value 4.2 × 10^− 6^, HER2+ predictors with p-value 1.1 × 10^− 5^**,** TNBC predictors with p-value 9.03 × 10^− 8^). Our results indicate that the predictors for breast cancer patients can also serve as prognostic markers for early stage breast cancer patients.

### Predictors for luminal, basal-like and HER2-enriched phenotypes and their prognostic power

In our predictive modeling analysis, we considered six different types of cells and generated a set of predictors which can accurately differentiate the six types. By following a more recent recommendation of classifying breast cancer, we categorized the cells into four groups: Luminal A (ER+), Luminal B (ER+ and HER2+), HER2-enriched, and Basal-like (TNBC). Then we followed the same procedure of removing genes with low expression, followed by removing genes with low variance across these four groups, and then followed by fitting a multinomial logistic regression to select important genes which selected 233 predictors out of which 23 were lncRNAs and 210 protein-coding genes (Additional file 1).

**Fig. 8:**
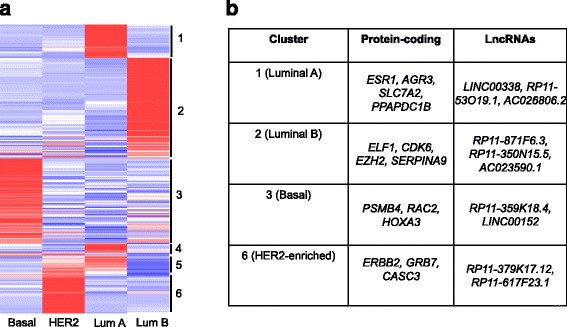
Clustering predictors for Luminal, Basal-like and HER2-enriched phenotypes. **a** Heat map representing the clustered expression profiles of 233 predictors for luminal, basal-like and HER2-enriched phenotypes. The cluster ID are indicated against the heat map. **b** Example protein-coding genes and lncRNAs belonging to the clusters are also shown

With these selected predictors, we tuned three models (support vector machines, neural network, and random forest) for accurate prediction of the four groups. We found that support vector machines yielded the best prediction accuracy (98.65%) followed by neural network (98.42% accuracy), and random forest (93.42% accuracy). To identify group-specific predictors, we further clustered these 233 predictors to using their expression profiles of these predictors. This procedure yielded 6 clusters (Fig. 8) consisting of predictors which are specific to luminal A (cluster 1, and 4), luminal B (cluster 2), HER2-enriched (cluster 6), and basal-like (cluster 3). Out of 233 predictors, 153 predictors (66%) were also found in our original 308 predictors for six different types of cells indicating common predictors between the two sets. Nevertheless, our results identify a set of predictors exist that accurately differentiate between Luminal A (ER+), Luminal B (ER+ and HER2+), HER2-enriched, and Basal-like (TNBC) subtypes of breast cancer.

## Discussion

In this study, we have successfully outlined a predictive analytics pipeline to accurately predict 6 breast cancer cell types using single cell gene expression profiles. The histological profiles and pathological examination of majority of the cells validated the labeled subtype, and strong correlation between cells derived from the same patient compared to cells from a different patient provided strong reasons that the designated cell type label was appropriate (GSE75688). The individual cells belonging to each type also clustered together (Additional file 1: Figure S2). We have showed that as a preprocessing step, a multinomial penalized logistic regression can be applied to retain only the important predictors. By using only this set of predictors, non-linear predictive models such as support vector machines and neural networks can accurately discriminate between the different cancer cell types. We also find that mixture of protein coding genes and long non-coding RNAs are better predictors compared to when the two sets of transcripts are treated separately. Additionally, we also highlight 6 clusters of genes that accordingly restricted to 6 different breast cancer cell types and validated the results by demonstrating several previously established breast cancer markers in each cluster. A signature risk score originating from 65 protein coding genes and 5 lncRNA predictors is associated with prognostic survival of TCGA breast cancer patients. This association was maintained when the risk scores were generated using 65 PCGs and 5 lncRNA separately. Therefore, a subset of the breast cancer cell type predictors is also associated with patient survivability and hence have clinical significance.

It is important to note that the penalized linear logistic regression process achieved an accuracy of 87%, but it yielded a set of 308 predictors to use for further analysis. Exploration with other non-linear models such as support vector machines and neural networks amplified the classification accuracy. This indicates that the cell type of a breast cancer cell can be expressed as dependent variable of expression profiles of a set of predictors; however, the relationship is nonlinear. The penalized logistic linear regression technique for feature selection process is appropriate for our study because it is computationally feasible on a data set with thousands of features. Other selection techniques such as wrapper methods are susceptible to over fitting and take a considerable amount of time to run and were not feasible for such king of data that contains high number of features. We were not able to find a different independent single cell sequencing data for breast cancer with known cell types to further validate our predictive model. Nevertheless, in our model building approach, we only used 80% of the entire data to generate the model. The remaining 20% was not used in the model building procedure and served as an “independent” validation set (Fig. 2).

There have been several studies reporting the prognostic power of protein coding genes in cancer. Recently, lncRNAs are also emerging as prognostic markers in different cancer including breast cancer. For the first time, our study reports lncRNAs which can discriminate different breast cancer cell types and also have prognostic potential. In our study, we found that the signature score of a set of 5 lncRNAs (ENSG00000250337, ENSG00000224137, ENSG00000266088, ENSG00000238121, and ENSG00000260257) can be used for breast cancer prognosis. Signature risk scores of sets of 12 lncRNAs [24], 9 lncRNAs [25], and 4 lncRNAs [26] have been reported by independent studies to have potential prognostic power in breast cancer patients. We found that none of the these previously reported sets overlap with our set of 5 lncRNAs. This shows that this set of 5 lncRNAs contribute to the discrimination of different breast cancer cell type as well have prognostic significance in breast cancer patients. Specific to breast cancer, there is a study that explored the oncogenic landscape of lncRNAs in breast cancer patients [27]. The study confirmed three lncRNAs which were subtype specific in the RNA-Seq results: *TINCR, LINC00511*, and *PPP1R26-AS1* represented the HER-2, triple negative and luminal B subtypes, respectively. They also reported that lncRNAs, *HOTAIR, LINC00115, MCM3AP-AS1, TINCR, PPP1R26-AS1*, and *DSCAM-AS1* were breast cancer prognosis-associated lncRNAs. These lncRNAs were absent in our set of predictors. The major differences between our study and this study is that we begin our analysis using a single cell RNA-Seq data, while the study from Xu et al., 2017 utilizes only cancer patient data. This study simply uses statistical methods of differential gene expression analysis to identify dysregulated lncRNAs in different subtypes of breast cancer. No machine learning predictive modeling was used in their approach to check if the dysregulated lncRNAs can accurately predict the subtype of cancer. In our approach, we performed analysis of variance (loosely similar to differentially expressed genes analysis) to lower the number of predictors followed by an optimal feature selection technique based on penalized regularized logistic regression to select a small set of predictors. We also employed machine learning models and clustering to validate that these genes and lncRNAs are not only good predictors of breast cancer subtypes but also can be grouped into subtype specific predictors. This shows that studying breast cancer using different methods can complement each other and are necessary in deciphering the underlying regulatory layers.

In summary, this study both validates the use of scRNA-seq to transcriptionally profile an ample number of cells originating from 6 different breast cancer cell types and defines 65 protein coding genes and 5 lncRNAs which are significantly related to prognostic survival of breast cancer patients from TCGA database. However, this pipeline cannot be applied only once as a variety of genetic and epigenetic changes has been implicated in the development and treatment of breast cancer. Heterogeneity in cancer patients is known to be dynamic and to evolve unpredictably during disease progression, which creates a significant challenge for modern cancer treatments. Several studies revealed evidences of instability of the hormonal and/or HER2 status during tumor progression, especially between primary tumor and metastatic tumors. Former studies have demonstrated that each intrinsic subtype has preferred chemotherapy regimen. For example, HER2 enriched type is expected to a sensitive response to anthracycline-based chemotherapy regimens, and basal like type is a response to platinum drugs. Our established predictive analytic pipeline has the potential to create a paradigm shift in cancer care to precision treatment where heterogeneity is thoroughly characterized prior to and during treatment. This approach may provide important new insights into cancer evolution and unveil new avenues for dissecting the complex activation of signaling pathways that cause heterogeneous cellular responses during treatment. Thus, we must reinforce the need for longitudinal characterizations of tumor transcriptomes by using our analytic pipeline to predict predominant breast cancer cell types so as to guide more personalized clinical care.

## Conclusions

Here, we outline a predictive analytics pipeline to accurately predict 6 breast cancer cell types using single cell gene expression profiles. Using machine learning techniques, we identify 308 predictors, out of which 34 are long non-coding RNAs, of breast cancer cell types. This set of predictor are able to identify different breast cancer cell types with 98% prediction accuracies. We also find that mixture of protein coding genes and long non-coding RNAs are better predictors compared to when the two sets of transcripts are treated separately. We further show that a signature risk score originating from 65 protein coding genes and 5 lncRNA predictors is associated with prognostic survival of TCGA breast cancer patients. This association was maintained when the risk scores were generated using 65 PCGs and 5 lncRNA separately. We further show that predictors restricted to a particular cell type serve as better prognostic markers for the respective patient subtype**.** Our results show that in general, the breast cancer cell type predictors are also associated with patient survivability and hence have clinical significance.

## Methods

The analysis pipeline used in this paper is summarized in Fig. 1 and is divided into two sections. The first part of the analysis is feature filtering and selection to reduce the number of gene predictors to be used in building subsequent predictive models. The second part of the analysis involves tuning various predictive models on the reduced data set. The single cell RNA-Seq data was obtained from NCBI GEO site (GSE75688 for Breast Cancer). The feature selection and predictive models are described in the next two subsections.

### Feature selection

Genes which had low expression across all samples (maximum expression < 2 FPKM or fragments per kilobase of exon per million reads mapped across all the samples) were first removed. Then, we performed analysis of variance (anova) on each of the remaining genes across the 6 cell types (groups) to test for significant effect of cell type on the expression. The *p*-value for each gene from this analysis and FDR values were then computed from the *p*-values [28]. The genes with FDR < 0.05 were only retained for further analysis.

A penalized logistic regression model with least absolute shrinkage and selection operator (LASSO) was trained using glmnet package [29]. Since the data set had multiclass cases, we used the “multinomial” option and grouped-lasso penalty on all the coefficients for particular variables [30]. LASSO retains one feature from a group of correlated features in the dataset, takes less computation time, and the feature selection process is embedded during the model training process. LASSO model was tuned by varying the value of regularization parameter lambda. To prevent over fitting, resampling of training set was done through 10-fold cross validation. The model with the least misclassification error was chosen. This optimal model also contained the best set of genes (or predictors).

### Prediction models

The set of genes or predictors selected by the optimal LASSO model was only retained in the data set for tuning subsequent predictive models. This reduced data set with fewer predictors was split into training (80%) and testing (20%) sets. To make sure that there was no bias in the split for a particular cell type; 80% of cells from each population contributed to training set and 20% of cells from each population contributed to testing set. Using 10-fold cross validation resampling technique, the following models were tuned by varying the respective model tuning parameters: K-nearest neighbor, decision trees, support vector machines, ensemble models (random forest and boosted trees), neural networks, and Naiive Bayes.

### Clustering and gene ontology

The clustering was done using the R HOPACH package [31] setting cosine dissimilarity as the distance metric. Gene ontology was performed using DAVID (32) by setting the human genome as the background.

### Ranking predictors

The ranking of the predictors were done using the VarImp function in CARET R package [23]. We used the model independent option while using the function. This method performs ROC curve analysis on each predictor. This area under the curve is used as the measure of variable importance.

### Statistical analysis

The univariate Cox regression analysis was performed to examine the relationship between the expression levels of breast cancer cell type predictors in TCGA breast cancer patients and the overall survivability from the training set with an aim to determine which predictors could potentially be of functional significance in breast cancer prognosis. Predictors that were significantly related to patient survival were identified (*p*-value ≤ 0.05) and then subjected to the multivariate Cox regression analysis. Each patient in the training set was assigned a risk score as the weighted sum of log2 expression values of the selected predictors (the weights were the coefficients obtained from the fitted multivariate Cox model). Based on the risk score, the patients in the training data set were divided into two groups - high risk (top one-half of signature risk score), and low risk (bottom one-half of signature risk score) patients. The patients in the testing data set were assigned a risk score using the same coefficients in the multivariate Cox model trained using the training data set. Differences in the overall survival between the two groups in both the training and testing sets were estimated and compared by the Kaplan–Meier method with a two-sided log-rank test.

## Additional file

Additional file 1: **Figure S1**, **Figure S2**, and **Table S1.** Prognostic potential of 6 sets of cell specific predictors in TCGA breast cancer patients, Cells belonging to the same type cluster together. (XLSX 801 kb)
